# In-depth evaluation of root infection systems using the vascular fungus *Verticillium longisporum* as soil-borne model pathogen

**DOI:** 10.1186/s13007-021-00758-x

**Published:** 2021-06-05

**Authors:** Christian Fröschel

**Affiliations:** Department of Pharmaceutical Biology, Julius-von-Sachs-Institute, Julius-Maximilians-Universität Würzburg, Julius-von-Sachs Platz 2, 97082 Würzburg, Germany

**Keywords:** *Arabidopsis thaliana*, *Brassica napus*, Indole-glucosinolates, Plant defence, Root infection systems, Root pathogens, Soil-borne microorganisms, *Solanum lycopersicum*, *Verticillium dahliae*, *Verticillium longisporum*

## Abstract

**Background:**

While leaves are far more accessible for analysing plant defences, roots are hidden in the soil, leading to difficulties in studying soil-borne interactions. Inoculation strategies for infecting model plants with model root pathogens are described in the literature, but it remains demanding to obtain a methodological overview. To address this challenge, this study uses the model root pathogen *Verticillium longisporum* on *Arabidopsis thaliana* host plants and provides recommendations for selecting appropriate infection systems to investigate how plants cope with root pathogens.

**Results:**

A novel root infection system is introduced, while two existing ones are precisely described and optimized. Step-by-step protocols are presented and accompanied by pathogenicity tests, transcriptional analyses of indole-glucosinolate marker genes and independent confirmations using reporter constructs. Advantages and disadvantages of each infection system are assessed. Overall, the results validate the importance of indole-glucosinolates as secondary metabolites that limit the *Verticillium* propagation in its host plant.

**Conclusion:**

Detailed assistances on studying host defence strategies and responses against *V. longisporum* is provided. Furthermore, other soil-borne microorganisms (e.g., *V. dahliae*) or model plants, such as economically important oilseed rape and tomato, can be introduced in the infection systems described. Hence, these proven manuals can support finding a root infection system for your specific research questions to further decipher root-microbe interactions.

**Supplementary Information:**

The online version contains supplementary material available at 10.1186/s13007-021-00758-x.

## Background

Many soil-borne microorganisms affect plant growth and cause disease. Within the fungal genus *Verticillium* (*Ascomycota*) some members are well-known plant pathogens that cause so-called vascular wilt diseases, leading to an estimated annual loss of €3 billion worldwide [1]. One important agent is *Verticillium longisporum*, which is highly adapted to brassicaceous hosts, such as oilseed rape (*Brassica napus*) and cauliflower [1]. Nowadays, oilseed rape is one of the top three sources of vegetable oil in the world and of economic importance by providing human food, animal feed and biofuels [2–4]. Depending on environmental conditions, yield losses in oilseed rape cultures caused by *V. longisporum* might locally range between 10 and 50% [1, 5, 6]. The infection process initiates in the soil from resting forms (microsclerotia), which germinate in response to plant exudates [7]. The fungal hyphae traverse the outer root cell-layers by intra- and intercellular growth and penetrate the vasculature in the inner central cylinder [1, 8]. Most of its life cycle, the fungus exists in the vascular tissue. There, the xylem sap is low in nutrients and distributes plant defence compounds, so that *Verticillium* spp. have to adapt to this unique environment by a fine-tuned secretion of colonization-related proteins enabling the pathogen to respond to changes in nutrient supply or host defences [9–11]. Once in the root vasculature, conidiospores may be released and disseminated acropetally with the water stream, leading to a systemic spread in the host and colonization of the foliage [1, 12]. From then on, the fungus affects plant growth [8, 9, 13], triggers premature senescence [14] and symptoms like stunting and yellow leaves occur [15, 16]. Thus, crop yield and quality of infected plants is strongly reduced. As up to date no efficient fungicides are available [1], further research is required to define genetic determinants of resistance to develop novel strategies for disease control in agriculture.

In response to pathogens, plants evolved inducible defence mechanisms. As one efficient strategy, plants produce secondary metabolites to defend themselves against invading microorganisms [e.g., 17]. The so-called tryptophan-derived secondary metabolites arise from the amino acid tryptophan (Trp). Among these, sulfur-containing camalexin and indole-glucosinolates (IG) are described in the model plant *Arabidopsis* to combat various plant pathogens [e.g., 18, 19]. Modifications in the biosynthetic pathway of these compounds, such as hydroxylation and subsequent methylation, require several CYTOCHROME-P450 enzymes (CYPs) (reviewed in [20]). The key enzymes in the beginning of the biosynthesis are CYP79B2 and CYP79B3. The double mutant *cyp79b2/b3* shows increased susceptibility to a variety of microorganisms, such as *V. longisporum* or *Phytophthora brassicae* [12, 21], which shows the importance of these secondary metabolites for defence. In *V. longisporum* infected *Arabidopsis* roots, gene expression of the IG pathway is heavily induced [12, 13]. A prominent example is the strong induction of *CYP81F2*, which encodes an important enzyme in the terminal part of IG biosynthesis. Recently, some transcription factors from the ETHYLENE-RESPONSE-FACTOR (ERF) family were shown to be activators of genes necessary for IG production [13, 22]. Several closely related ERFs (group IX) enhance resistance against *V. longisporum* when overexpressed. One example is ERF105, which directly binds to and activates the *CYP81F2* promoter [13].

To investigate interactions of roots with soil-borne microorganisms, several studies combine *Arabidopsis thaliana* and *V. longisporum* [12–14, 16]. Central reasons for using these models are easy-to-handle susceptibility assays and well-established genetic resources to study expressional adaptations and regulatory mechanisms. Through comparative analysis of the molecular events explained above, we optimized two existing infection systems and developed one new in vitro infection system. We highlight advantages and disadvantages of each infection system to help identify an appropriate method for specific research questions in general root-microbe interactions.

## Material and methods

### Material

The virulent *Verticillium longisporum* (*Vl*) strain *Vl*43, originally isolated from *Brassica napus* in northern Germany [23, 24], was chosen for this study. It possesses a cefotaxime resistance. *Vl*-sGFP (i.e., *Vl*43 constitutively expressing enhanced Green Fluorescent Protein; [8]) was used as fluorescent fungal reporter line. *Verticillium dahliae* (*Vd*) isolate JR2 (originally isolated from tomato, but it also infects *Arabidopsis* under standard laboratory growth conditions [25, 26]) possesses cefotaxime resistance and was obtained from [26]. *Arabidopsis thaliana* wild type (WT) and all transgenic lines had Col-0 (Columbia) background: *cyp79b2/b3* [27], *ERF105* overexpressing line (*ERF105 OE*) [13], *Promoter*_*CYP79B2*_*:LUC* [12]. WT oilseed rape (*Brassica napus* var. *napus*) was from the cultivar “Miniraps” (rapid-cycling rape genome ACaacc; [9, 28]). WT tomato (*Solanum lycopersicum*) was used as an additional model plant.

### Surface sterilization of seeds

Seeds from *A. thaliana*, *B. napus* and *S. lycopersicum* were surface sterilized by incubation with chlorine gas for 3 h prior to sowing. Chlorine gas was generated in a desiccator containing the seeds through the reaction of 100 ml of 12% sodium hypochlorite (NaClO in H_2_O) and 6 ml of 33% hydrochloric acid (HCl).

### Cultivation of *V. longisporum* and inoculum preparation

Note, all equipment and medium had to be germ-free. Additionally, all steps were performed in a laminar flow hood to keep the inoculum clean. To obtain mitotically derived (asexual) conidiospores, the mycelium was cultured in liquid Potato Dextrose Broth medium (PDB; Sigma-Aldrich GmbH, Germany) supplemented with 500 mg/l cefotaxime (Duchefa, The Netherlands). A 500 ml chicanery flask was filled with 150 ml PDB medium and inoculated with *Vl*43 conidiospores from a glycerol-stock storage. 7–10 days incubation in darkness at room temperature (RT) under continuous, horizontal shaking (rotary shaker, 60 rpm) resulted in small, spherical, white mycelia (Additional file 1: Figure S1a, b). The PDB medium was carefully removed, while the mycelia remained in the flask. To induce sporulation, 100 ml of Czapek Dextrose Broth medium (CDB, Duchefa) supplemented with 500 mg/l cefotaxime was added to the mycelia. Another 4–5 days incubation in darkness at RT under continuous, horizontal shaking (rotary shaker, 60 rpm) resulted in a yellow-greyish supernatant (Additional file 1: Figure S1c) containing the conidiospores. To separate the spores from the mycelia, a portion of the supernatant was filtered through filter paper (pleated cellulose filter, particle retention level 8–12 µm) into a sterile 50 ml collection tube (Additional file 1: Figure S1d, e). By using a Thoma cell counting chamber, the spore-concentration was determined and diluted with germ-free ¼ MS (Murashige & Skoog medium including vitamins, Duchefa) in Milli-Q water until the final spore-concentration was achieved (details see below). Freshly harvested conidiospores were always used as inoculum.^1^ For long-term storage, isolates were frozen as high concentrated spore solutions (approx. 1 × 10^8^ spores/ml) in 25% glycerol at − 80 °C. 100 µl of these glycerol stocks were used to inoculate the PDB medium in the beginning of this section.

Cultivation and inoculum preparation of *V. dahliae* (JR2) was done in exactly the same manner as for *V. longisporum*.

### Sterile in vitro infection system in petri dishes

The petri dish cultivation system based on a medium consisting of 1.5 g/l MS (Murashige & Skoog medium including vitamins, Duchefa) and 8 g/l Gelrite Agar (Carl Roth GmbH, Germany) in Milli-Q water.^2^ After autoclaving (121 °C), the medium was poured into petri dishes (92 × 16 mm, Sarstedt AG & Co. KG, Germany). All steps were performed in a laminar flow hood with germ-free equipment. The upper third as well as an infection channel were cut and removed with a scalpel from the solidified medium^3^ as illustrated in Additional file 1: Figure S1f. Per plate, 50–100 surface sterilized *Arabidopsis* seeds (former protocols [12, 13] suggest 30–40 seeds per plate. We recommend to use more to get more root material facilitating down-stream approaches) were distributed with a pipette tip on the cut surface directly at the angle to the petri dish wall (enabled roots to grow between medium and petri dish wall, which facilitated inoculation and harvesting compared to a root growth completely in medium). The petri dishes were sealed with Leukopor® (BSN medical GmbH, Germany) allowing gas exchange. After stratification for 2 d in darkness at 4 °C, the plates were set up vertically and plants were grown at 22 °C ± 1 °C under long day conditions (16 h light/8 h darkness) in a poly klima® growth chamber (poly klima GmbH, Germany). As soon as the roots reached the channel (9–11 days old seedlings), 500 µl of freshly (see footnote 1) harvested *V. longisporum* (*Vl*43) conidiospores (4 × 10^5^ spores/ml) were added to the infection channel (Additional file 1: Figure S1g). To prepare control plates, 500 µl mock solution without spores was used. After adding the solutions, the plates were incubated horizontally in the laminar flow hood for at least 40 min before they were sealed with Leukopor®. The root parts were covered with black paper-boxes and plates were placed vertically again (Additional file 1: Figure S1h). To control success of infection, it was demonstrated by microscopy that the shape of infected roots differed from mock treated ones (Additional file 1: Figure S1i). At the time points indicated, leaves were cut from the roots and harvested separately. By taking the agar strips out of the petri dishes, roots became easily accessible and were carefully pulled out of the agar using forceps. All material was flash-frozen in liquid nitrogen and used either for analyses of transcriptional changes or determination of fungal DNA. Each biological replicate consisted of pooled leaves or roots from two plates.

### Soil-based infection system in pots

This system could be kept semi-sterile. Whereas previous literature [12, 16, 26] describes to use a 1:1 (v/v) soil:sand mixture on Seramis, our study used a 3:1 (v/v) soil:sand mixture (bird sand from Pet Bistro, Müller Holding Ltd. & Co. KG, Germany) without Seramis. The higher soil content enabled a better growth and pre-cultivation of the plants, while there was still enough sand to properly excavate the roots later. After steaming^4^ the soil:sand mixture, it was filled into pots (Ø 7 cm). The pots were transferred to trays and water was added into the trays about 1/3 the height of a pot. 3 h later the soil/sand mixture was thoroughly soaked with water. In addition, the soil surface was water-sprayed with a spray bottle, to ensure wet starting conditions. 3–4 seeds were sown per pot and stratified for 3 d in darkness at 4 °C to synchronize germination. Thereafter, plants were cultivated for 21 days under long-day conditions (16 h light / 8 h darkness; 22 °C; 60% humidity) and regular watering.^5^ To reduce individual variations, an excess of plants was pre-cultivated and just plants of similar size were carefully selected for the actual experiments. Additional file 1: Figure S2a and b illustrate the procedure, called “root dip inoculation” [12, 16]. 21 days old *Arabidopsis* plants were prepared: after taking the soil out of the pots, roots were carefully excavated, gently washed in a water container^6^ and dipped for 60 min in a petri dish holding 35 ml of *Vl*43 spore-solution (2 × 10^6^ spores/ml) or a mock solution without spores. At this point, the protocol of preceding studies [12, 16, 26] (dipping for 45 min in 1 × 10^6^ spores/ml) was modified as this—in our hands—improved subsequent infection success, reliability in pathogenicity tests, and reproducibility. Following the 60 min incubation, plants were inserted^7^ into fresh single pots containing moist, steam-sterilized soil (see footnote 4) without sand and cultivated under long-day conditions (16 h light / 8 h darkness; 22 °C; 60% humidity) with regular watering (see footnote 5). Only the rosettes were harvested by cutting them at the root crown at the time points indicated. To analyse fungal DNA, rosettes of 5 plants were pooled per replicate and at least 3 biological replicates were analysed per line. All material was flash-frozen in liquid nitrogen.

### Sterile in vitro infection system in plastic cups

A novel sterile in vitro system in plastic cups was established. All steps were carried out in a laminar flow hood with germ-free equipment. All plastic ware was sterilized in a 70% ethanol bath for at least 20 min. Additional file 1: Figure S3a-h illustrate the whole principle of this infection system. In brief, a MS (Murashige & Skoog medium including vitamins, Duchefa) based Gelrite Agar (Carl Roth GmbH) medium (see footnote 2) (4.4 g/l MS, 0.2 g/l MgSO_4_, 1 g/l KNO_3_, 0.5 g/l MES [2-(*N*-morpholino)ethanesulfonic acid], 6.0 g/l Gelrite in Milli-Q water, adjust pH 5.7 with KOH) was autoclaved (121 °C) and poured into transparent 500 ml plastic cups (108 × 82 × 99 mm, salad boxx® Feinkostbecher).^8^^,^^9^ A separating plastic layer was transferred to the medium before it solidified. It contained holes for placing surface sterilized seeds onto the medium. This allowed the seeds an access to the medium and later it prevented the leaves from touching the fungus-containing medium. Therefore, all fungal DNA detectable in leaves derived solely from fungal spread within the plant (from root to shoot). As soon as the medium solidified, seeds were transferred to the prefabricated holes (important note^10^) and an approx. 1.5 cm deep infection channel was cut through a centre hole, which later enabled the addition of fungal spores. The cups were covered with a second, inverted plastic cup and sealed with Leukopor® (BSN medical GmbH).^11^ To synchronize germination, seeds were stratified for 3 days in darkness at 4 °C. Seedlings were grown under 12 h light/12 h darkness conditions in percival chambers (CLF Plant Climatics GmbH, Germany) at constant 22 °C and 60% humidity. In case of *Arabidopsis*, 21 days old plantlets were inoculated with *V. longisporum* (*Vl*43) by adding 1 ml conidiospore solution (4 × 10^5^ spores/ml) into the infection channel cut in the beginning. For control samples, 1 ml mock solution without spores was added. The whole rosettes were harvested at the time points indicated and prepared to analyse the amount of fungal DNA or expressional changes. Roots were carefully pulled out of the medium, squeezed and dabbed with a paper towel to remove agar residues. Each biological replicate consisted of rosettes / roots pooled from 5 plants and at least 3–4 biological replicates were analysed per line or time-point. All material was flash-frozen in liquid nitrogen.

*Brassica napus* was treated with *Vl*43 similar to *Arabidopsis*, but as this species grows faster, 5–7 days old seedlings were inoculated. At the time points indicated, all leaves present on each *B. napus* plant were harvested and leaves from 5 plants were pooled for each replicate. All *B. napus* material was flash-frozen in liquid nitrogen to determine fungal DNA.

For the experiments analysing *V. dahliae* (JR2) propagation, 21 d old *Arabidopsis* plantlets (grown under 12 h light/12 h darkness conditions, constant 22 °C, 60% humidity) or 12 d old tomato plants (grown under 16 h light/8 h darkness conditions, constant 22 °C, 60% humidity) were inoculated by adding 1 ml of a JR2 conidiospore solution (4 × 10^5^ spores/ml) into the infection channel. Whole *Arabidopsis* rosettes were harvested 12 dpi as described above and tomato stem segments were cut 14 dpi. Again, material from 5 plants was pooled for each replicate and 3–4 biological replicates were prepared. All plant material was flash-frozen in liquid nitrogen to analyse the amount of fungal DNA.

### Quantification of* Verticillium* DNA via quantitative PCR (qPCR)

The amount of *Verticillium* DNA was quantified in leaves to estimate fungal propagation *in planta*. After grinding the leaf material in liquid nitrogen, 100 mg powder was transferred to a 2 ml reaction tube and used to extract total DNA (CTAB [cetyltrimethylammonium bromide] method for DNA extraction modified from [29]: 400 µl CTAB extraction buffer was added to each sample, followed by vortex and a 15 min incubation at 65 °C. 400 µl chloroform:isoamyl alcohol (24:1, v/v) was added, followed by vortex and centrifugation at 13,000 rpm for 5 min at RT. 280 µl of the aqueous layer was transferred to a clean tube and 280 µl isopropanol was added. It was mixed by inversions and incubated for 3 min at RT. It was centrifuged at 13,000 rpm for 10 min at RT. The supernatant was discarded and the pellet was washed with 300 µl cold 70% ethanol. A brief centrifugation secured the pellet so that the supernatant could be carefully removed with a pipette. The pellet was dried and the DNA was dissolved in water. The concentration was adjusted to approx. 50 ng/µl. qPCR was performed with 100 ng total DNA and BIOTAQ™ DNA polymerase (Bioline GmbH, Germany) in a CFX96™ Real-Time PCR Detection System (Bio-Rad Laboratories, Inc., USA). Amplification products were visualized by SYBR® Green (Lonza Group AG, Switzerland). These conditions were generally applied: 10 min at 95 °C, 40 cycles of: 20 s at 95 °C, 20 s at 56 °C and 20 s at 72 °C, followed by a quality check program determining the melting curve to detect nonspecific amplification. Fungal DNA (both from *V. longisporum* and *V. dahliae*) was determined using *Verticillium* specific primers OLG70 and OLG71 [8] and evaluated relative to an *ACTIN8* (*ACT8*) [13] amplicon derived from *Arabidopsis thaliana* or *Brassica napus* genomic DNA or relative to a *RuBisCo* (*RUB*) amplicon derived from *Solanum lycopersicum* genomic DNA [30]. Quality control was performed showing specificity of the primers depending on DNA type (Additional file 1: Figure S4). Relative amount of fungal DNA was calculated as x-fold over WT or mock by 2^−ΔΔCT^ method [31]. One data point was calculated from 3–5 biological replicates (*n*). Primer sequences are given in Additional file 1: Table S1. Determination of fungal DNA from root material is difficult and was not done here, as you have to distinguish whether the mycelium has grown outside or inside the roots to analyse host susceptibility.

### Detection of transcriptional responses via quantitative reverse transcription PCR (qRT-PCR)

Each RNA sample was isolated from roots or leaves following the TRI Reagent procedure [32]. 30 min of DNase I treatment (Thermo Fisher Scientific, Inc.) was performed to eliminate DNA contaminations in RNA extracts. cDNA was synthesized from 1 µg of total RNA using oligo(dT) primers, random nonamer primers and the reverse transcriptase RevertAid H Minus (Thermo Fisher Scientific, Inc., USA) according to the manufacturer’s manual. 2 µl of 1:10 diluted cDNA and BIOTAQ™ DNA polymerase (Bioline GmbH) were used in a CFX96™ Real-Time PCR Detection System (Bio-Rad Laboratories, Inc.). Amplification products were visualized by SYBR® Green (Lonza Group AG). This conditions were generally applied: 10 min at 95 °C, 40 cycles of 20 s at 95 °C, 20 s at 56 °C and 20 s at 72 °C, followed by a default dissociation stage program to detect nonspecific amplification. Primers are given in Additional file 1: Table S1. Values were calculated from at least 3 biological replicates as fold induction values using 2^−ΔΔCT^ method [31]. *UBQ5* served as reference gene for normalization.

### Analysis of green leaf area and fresh weight from *Arabidopsis*

After removing the stems, photographs of the plants were taken with a digital camera always at the same distance from above. Projected green leaf area was assessed by using *BlattFlaeche*® (Datinf GmbH, Tübingen, Germany) software as described in [12] and Additional file 1: Figure S5a. The diameter of the culture pots was used as an internal standard for normalizing the leaf area. Biomass (fresh weight) of *Arabidopsis* rosettes was determined after removing roots and stems by weighing whole rosettes. Relative fresh weight was determined by normalizing fresh weight of infected samples to that of the corresponding mock samples.

### Analysis of fungal colonization in stems

21–30 dpi (days post inoculation), 1–1.5 cm of primary inflorescence stem segments were cut at the base (Additional file 1: Figure S5b), surface sterilized (5 min, 70% ethanol; 5 min 0.02% sodium hydrogen chloride solution supplemented with 0.02% Triton X-100) and thoroughly washed with germ-free water (according to [13]). These stem segments were placed on solidified Potato Dextrose Broth agar (20 g/l PDB (Sigma-Aldrich); 10 g/l phyto agar (Duchefa); Milli-Q water; autoclaved at 121 °C; supplemented after cooling to 60 °C with 500 mg/l cefotaxime (Duchefa). After incubation in darkness for 3–5 days at RT, fungal mycelium became visible.

### Fluorescence confocal microscopy

*Arabidopsis* was inoculated in the petri dish system with *Vl*-sGFP conidiospores. Roots were transferred to microscope slides, water incubated and covered with coverslips. Examination was achieved using Leica TCS SP5 II confocal laser scanning microscope (Leica Camera AG, Germany). Samples were excited by Argon Ion Laser and the fluorescence signal for GFP was detected (excitation wavelength: 488 nm; detection window: 500–530 nm). Pictures were taken with a HC PL APO 20 × 0.70 IMM CORR CS water immersion objective. For propidium iodide (PI) staining, roots were incubated in darkness for 10 min in 15 μM PI (P4170, Sigma-Aldrich GmbH). Thereafter, roots were transferred to microscope slides, incubated in water and carefully covered with coverslips. PI fluorescence signal was detected (excitation 488 nm, detection window 610–670 nm) and merged with GFP channel.

### Luciferase assay

*Arabidopsis* reporter line *Promoter*_*CYP79B2*_*:LUC* was inoculated with *V. longisporum* in the petri dish system. Roots were sprayed (3 dpi) with Luciferin solution (1 mM Luciferin, 0.02% Triton-X 100, demineralized H_2_O). Plates were incubated in a dark box of the luciferase imaging system C4742-98 coupled with cooled CCD camera (Hamamatsu Photonics K.K., Japan). Black/white and luminescence pictures were taken. Intensities of luminescence were given in false colours (low intensity in blue; high intensity in red).

### Statistical analysis

Calculations and graphs were made with Microsoft® Excel software. Statistical significance was assessed by using two-tailed Student’s *t*-test. Differences between two groups were considered significant with * *p* ≤ 0.05, ** *p* ≤ 0.01 or *** *p* ≤ 0.001. Error bars indicate ± SD (standard deviation) or ± SEM (standard error of the mean) as indicated in the figures.

### Gene accessions

*ACTIN8* (At1g49240); *CYP79B2* (At4g39950); *CYP79B3* (At2g22330); *CYP81F2* (At5g57220); *ERF105* (At5g51190); *UBQ5* (At3g62250).

## Results

In three different infection systems, roots were inoculated with *V. longisporum* and the host defence responses were compared. As it is known that IG are important to limit *V. longisporum* spread [13], expression of prominent marker genes (*CYP79B2*, *CYP79B3*, *CYP81F2* and *ERF105*) was analysed in mock treated and infected plant samples using qRT-PCR. The reliability of the systems in detecting variances in infection progress was tested employing a knockout mutant in the IG biosynthesis (*cyp79b2/b3*) and a transgenic line overexpressing a transcriptional activator (*ERF105*) in the IG pathway. The degree of fungal colonization in hosts was classified by determining the amount of *V. longisporum* DNA in leaves through qPCR analyses.

### The petri dish system is best suited to study early expressional responses

**Fig. 1:**
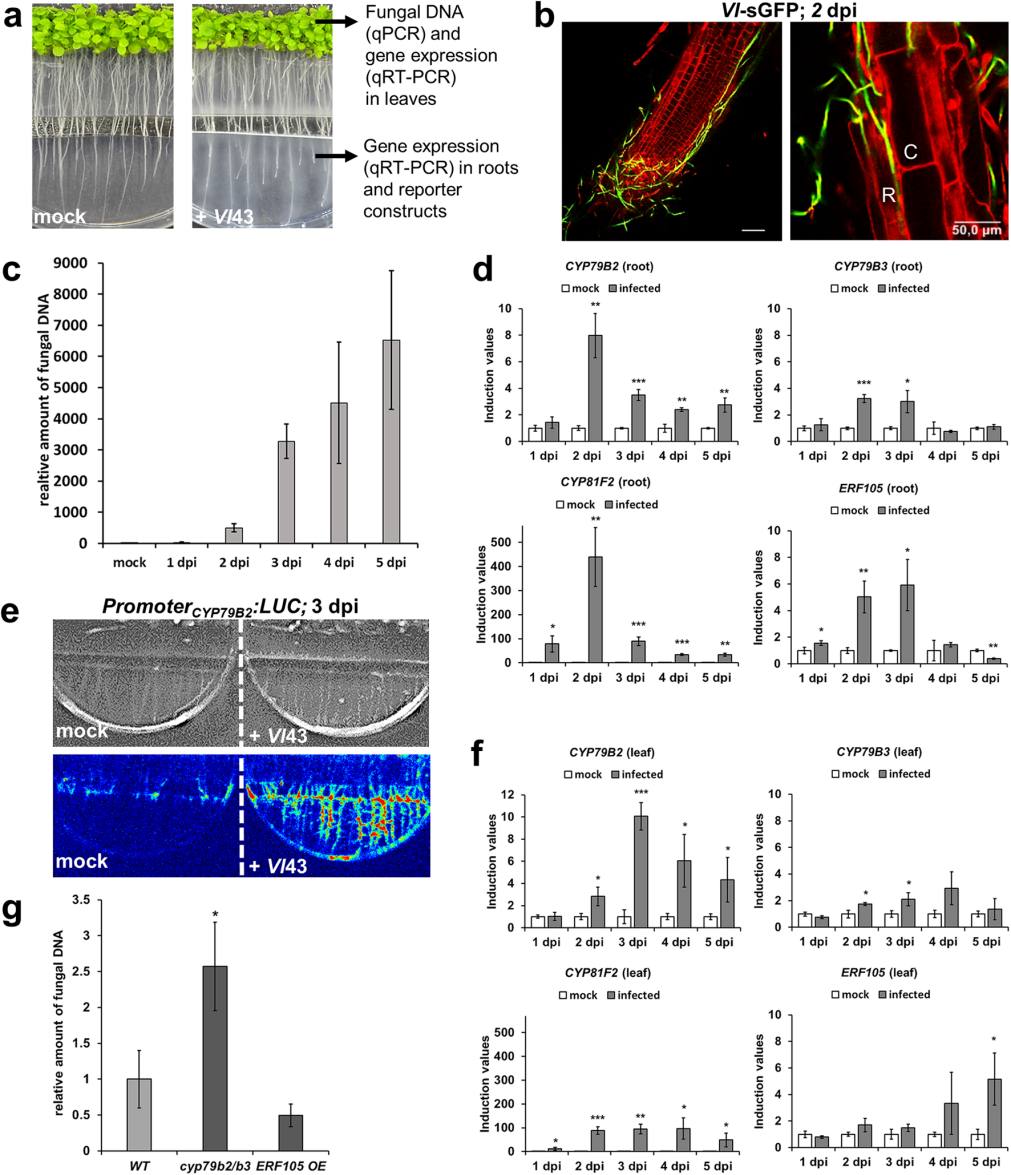
Infection system in petri dishes to study early *Arabidopsis-Verticillium* interactions. **a** Visible growth of *V. longisporum* mycelium 3 days post inoculation (dpi). Representative pictures from inoculated (+*Vl*43) and mock treated samples (adapted from [33]). Possibilities for downstream analyses are given. **b** Visualization of fungal growth by using a GFP-tagged *V. longisporum* strain (green, 2 dpi). Counterstaining of root-cells with propidium iodide (red). A net of fungal hyphae around the root tip (left) and hyphal growth between cells of rhizodermis (R) and cortex (C) (right). Bars specify 50 µm. **c** Relative amount of *Vl*43 fungal DNA as quantified in WT leaves at the time points indicated. Values of infected samples are given relative to background noise in mock samples (set to 1) (*n* = 3 each, ± SD). **d** Induction of marker genes in WT roots at the indicated time points. Values of *Vl*43 infected samples are given relative to mock samples (set to 1) (*n* = 3 each, ± SD). **e** The luciferase reporter line *Promoter*_*CYP79B2*_*:LUC* shows an extensive activation of *CYP79B2* promoter by *Vl*43 (3 dpi) compared to mock. Upper panel: black/white pictures of the system. Lower panel: intensities of luminescence are given in false colours from low (blue) to high (red). **f** Induction of marker genes in WT leaves at the indicated time points. Values of *Vl*43 infected samples are given relative to mock samples (set to 1) (*n* = 3 each, ± SD). **g** Amount of *Vl*43 fungal DNA in WT, *cyp79b2/b3* and *ERF105 OE* as quantified in leaves, 2 dpi. (*n* = 3 each, ± SD). This figure statistics: student ‘s *t*-test relative to mock (**d**, **f**) or WT (**g**), * *p* ≤ 0.05, ** *p* ≤ 0.01, *** *p* ≤ 0.001

Previously, a system to analyse whole root transcriptional responses against *V. longisporum* was established [12]. In our study, it was slightly improved (see “Materials and methods”). Soon after inoculation with conidiospores at roots, fungal mycelium became visible surrounding the roots and the adjacent medium (Fig. 1a). Fungal hyphae appeared all around the root tip and penetration of the outer root tissue was observed using a GFP-tagged *V. longisporum* strain (*Vl-*sGFP) (Fig. 1b). Fungal DNA was detectable in leaves as early as 1 to 2 days post inoculation (dpi), which indicates a rapid systemic spread in this infection system (Fig. 1c). An almost exponential fungal propagation occurred until 3 dpi, followed by a flattening of the curve. In roots, strong induction of IG marker genes was observed, displaying a maximum value at 2–3 dpi (Fig. 1d). Beyond the induction maxima, expression decreased consecutively, suggesting a role of IG especially during early infection. A luciferase reporter construct confirmed activation of the *CYP79B2* promoter in infected roots (Fig. 1e). Root and leaf responses can easily be examined separately in this system. Induction of IG marker genes in leaves reached the maximum at 3 dpi or even later (Fig. 1f), thus slightly delayed compared to root responses. The knockout mutant *cyp79b2/b3* lacks IG production [27] and it was significantly more susceptible than the WT. In contrast, the *ERF105* overexpressor (OE) tended to be more tolerant in this infection system, although not significant (Fig. 1g). Overall, this system is well suited to examine early expressional responses in both roots and leaves, but it has its limitations in assessing susceptibility/tolerance of plant genotypes.

### The soil-based system is well suited for susceptibility assays

**Fig. 2:**
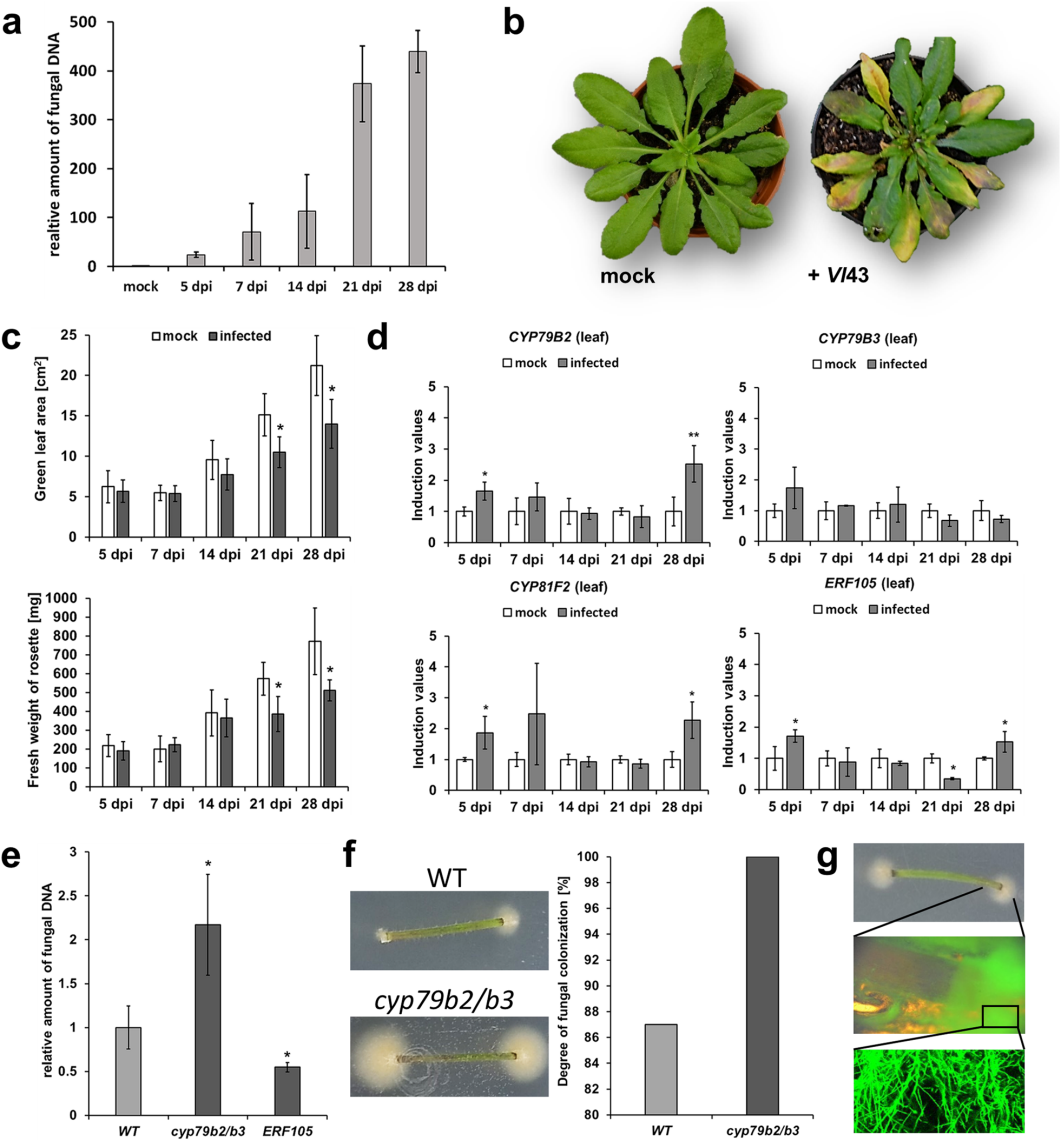
The soil-based infection system to study fungal propagation and leaf responses. **a** Relative amount of *Vl*43 fungal DNA as quantified in WT leaves at the time points indicated. Values of infected samples are given relative to background noise in mock samples (set to 1) (*n* = 3 each, ± SD). **b** Comparison of *Vl*43 infected and mock treated plants, 21 dpi. **c** Development of disease symptoms as quantified by green leaf area (upper part) and biomass (fresh weight, lower part) for whole rosettes of mock treated and *Vl*43 infected WT plants (*n* = 8 each, ± SD). **d** Induction of marker genes in *Arabidopsis* WT leaves at the indicated time points. Values of *Vl*43 infected samples are given relative to mock samples (set to 1) (*n* = 3 each, ± SD). **e** Amount of *Vl*43 fungal DNA in WT, *cyp79b2/b3* and *ERF105 OE* as quantified in leaves, 21 dpi. Values are given relative to WT (set to 1) (*n* = 3 each, ± SD). **f** Analysis of fungal colonization in stems of *Arabidopsis*, 21 dpi. 100% of the *cyp79b2/b3* and 87% of the WT stem segments were severely colonized with *V. longisporum* (100 stem segments tested in each case). Stem segments of *ERF105 OE* are less colonized than WT as recently published [13]. **g** Confirmation of *V. longisporum* outgrowth by using *Vl*-sGFP: stem segment with outgrowth (top), enlargement of stem end and outgrowth under fluorescence excitation (middle) and enlargement of the fluorescent hyphae (below). This figure statistics: student ‘s *t*-test relative to mock (**c**, **d**) or WT (**e**), * *p* ≤ 0.05, ** *p* ≤ 0.01

Since Koike et al. [34] published a technique for successfully initiating *Verticillium* disease in the greenhouse by dipping roots into spore suspensions, this protocol has been frequently modified, including the adjustments in this work (see “Materials and methods”). Approximately one week after root inoculation with *V. longisporum*, fungal DNA was detectable in *Arabidopsis* leaves and its amount increased over time (Fig. 2a). From 21 dpi on, leaves turned yellow and other symptoms such as stunting and reduced rosette size occurred (Fig. 2b and Additional file 1: Figure S2b, c). Due to the development of disease symptoms, green leaf area and biomass (fresh weight) of plant rosettes were significantly lower in infected WT than in mock treated WT at 21 and 28 dpi (Fig. 2c). Interestingly, when analysing gene expression in leaves, IG marker genes were less up-regulated than in the other infection systems. They were just slightly induced at the beginning (5 dpi) and the end (28 dpi) of the time course (Fig. 2d). However, relative fresh weight from infected *cyp79b2/b3* plants was significantly less than from infected WT (Additional file 1: Figure S2d), although the difference was not dramatic. Disparities became more obvious through quantification of fungal DNA. In line with the results above, the *cyp79b2/b3* knockout mutant had significantly more and the *ERF105 OE* significantly less fungal DNA in rosette leaves compared to WT (Fig. 2e). An alternative procedure to categorize susceptibility is quantifying fungal outgrowth from stem segments cut from infected plants [13] (Additional file 1: Figure S5b). In agreement with the other results, there was more fungal outgrowth from *cyp79b2/b3* stems than from WT stems (Fig. 2f). A control experiment using *Vl*-sGFP verified that it is indeed *V. longisporum* growing out of the stems (Fig. 2g). Taken together, this system works well for susceptibility testing using both symptom development, stem colonization, and the amount of fungal DNA as parameters.

### A novel set-up with plastic cups closes experimental gaps of other systems

**Fig. 3:**
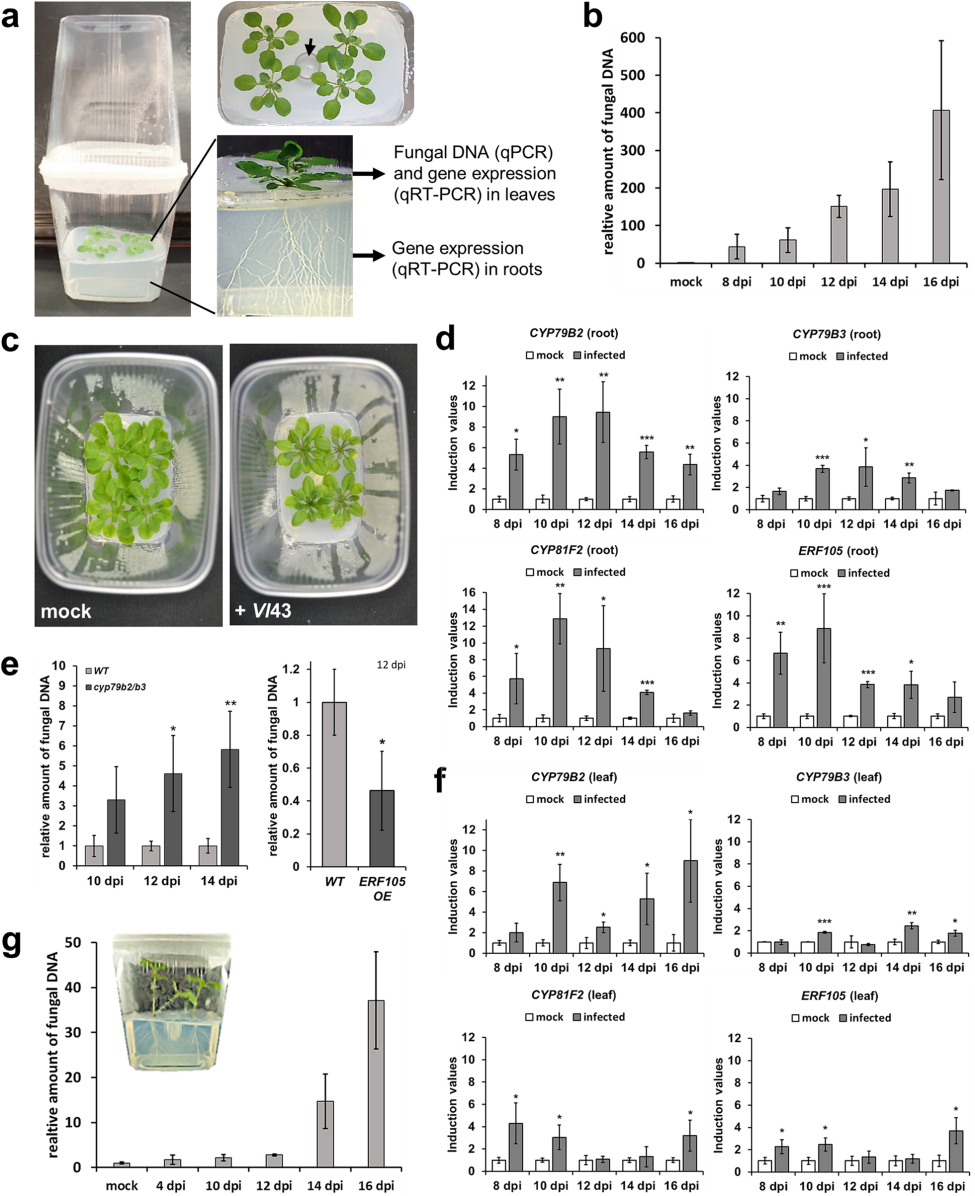
The in vitro infection system in plastic cups discloses root and leaf responses as well as fungal propagation. **a** Basic set-up of the system: four plants grow on agar medium in the lower plastic cup, while an inverted cup serves as lid. Conidiospores are added in a channel in the middle of the plants (black arrow). A separating plastic layer on the agar medium prevents a direct contact of the leaves with *V. longisporum* containing agar. Therefore, all fungus detectable in leaves results from spread within the plant. Possibilities for downstream analyses are given. **b** Relative amount of *Vl*43 fungal DNA in *Arabidopsis* WT leaves at the time points indicated. Values of infected samples are given relative to background noise in mock samples (set to 1) (*n* = 3 each, ± SD). **c** Representative photos from mock treated and *Vl*43 infected *Arabidopsis* WT plants, 12 dpi. **d** Induction of marker genes in *Arabidopsis* WT roots at the indicated time points. Values of *Vl*43 infected samples are given relative to mock samples (set to 1) (*n* = 3 each, ± SD). **e** Relative amount of *Vl*43 fungal DNA as quantified in *Arabidopsis* leaves: in WT and *cyp79b2/b3* at the time points indicated (left) and in WT and *ERF105 OE*, 12 dpi (right). All values are given relative to WT (set to 1) (*n* = 3 each, ± SD). **f** Induction of marker genes in *Arabidopsis* WT leaves at the indicated time points. Values of *Vl*43 infected samples are given relative to mock samples (set to 1) (*n* = 3 each, ± SD). **g** Relative amount of *Vl*43 fungal DNA as quantified in *Brassica napus* WT leaves at the time points indicated. Values of infected samples are given relative to background noise in mock samples (set to 1) (*n* = 3 each, ± SD). Inlay: cup with *B. napus*. This Figure statistics: student ‘s *t*-test relative to mock (**d**, **f**) or WT (**e**), * *p* ≤ 0.05, ** *p* ≤ 0.01, *** *p* ≤ 0.001

Although the petri dish system is good for assessing differentially expressed genes, it has weaknesses in pathogenicity assays and vice versa the soil-based system. Therefore, we established a new system combining advantages of both. Its reliability was assessed by comparing the results with those obtained from the two other infection systems. Figure 3a briefly summarizes the main features. A plastic cup harbours the plants in agar medium and an inverted cup is used as a lid. Four plants were cultivated per cup and inoculated with *V. longisporum* trough an infection channel located in the middle of the agar medium. Nicely, the fungus grew towards the roots so that a uniform infection of all plants was achieved. A time course corroborated that the fungus reached the foliage of *Arabidopsis* after root inoculation (Fig. 3b). Fungal DNA was first detectable in leaves 4 dpi (not shown) and substantially increased until 16 dpi. At later time points, the curve flattened (not shown). Subtle disease symptoms occurred on leaves, such as yellowing and reduction in rosette size (Fig. 3c). Marker genes for IG biosynthesis were transcriptionally induced in roots, as demonstrated in a time-course experiment: after reaching a maximum at 10–12 dpi, expression decreased again (Fig. 3d). This observation was similar to the petri dish system but time-delayed. By comparing WT with the highly susceptible *cyp79b2/b3* mutant, it turned out that 10 dpi was too early for sensing significant differences in susceptibility. Between 12 and 14 dpi significantly more (four to sixfold) fungal DNA was observed in the mutant (Fig. 3e). In contrast, *ERF105 OE* possesses increased IG marker gene expression [13]. This line was more tolerant than WT, since less fungal DNA and more relative fresh weight were found for the overexpressor upon infection (Fig. 3e and Additional file 1: Figure S3i). Transcriptional induction of IG markers was also observed in leaves. Maxima occurred at the beginning and at the end of the time-course (Fig. 3f), a pattern similar to that in the soil-based system.

To test applicability of other model plants in this system, *Brassica napus* plantlets were root inoculated with *V. longisporum*. The amount of fungal DNA in leaves of *B. napus* also increased with time (Fig. 3g). While fungal propagation was slow in the beginning of infection, it rapidly increased from 12 dpi on.

To evaluate whether infection assays with other soil-borne microbes are feasible in this system, roots were inoculated with the pathogen *Verticillium dahliae* that is closely related to *V. longisporum*. Both species perform a quite similar vascular life-style, but with different efficiency and distinct disease symptom patterns at *Arabidopsis*: *V. longisporum* typically induces early senescence, while *V. dahliae* infection causes wilting [26]. We analysed amount of *V. dahliae* DNA in leaves of *Arabidopsis* (12 dpi) and hypocotyls of tomato (14 dpi). By comparing with mock treated controls, it was confirmed that this fungus also propagates in both plant species from the roots to the above ground plant parts applying this infection system (Additional file 1: Figure S6). Furthermore, *Arabidopsis cyp79b2/b3* plants had significantly more and *ERF105 OE* significantly less fungal DNA than WT, suggesting that Trp-derived secondary metabolites have an impact against *V. dahlia*e (Additional file 1: Figure S6b, c).

In a nutshell, the novel infection system in plastic cups is well applicable and reliable for studying *Verticillium* ssp. diseases in the general model plant *Arabidopsis* and the agronomically interesting model plants *Brassica napus* and tomato.

## Discussion

This comparative study revealed advantages and disadvantages of three versatile infection systems with the vascular root pathogen *Verticillium longisporum*. It may assist scientists in finding appropriate infection systems for specific research questions.

### Advantages and disadvantages of the infection system in petri dishes

This easy-to-handle set-up leads to results within 2 weeks. During the whole procedure, the system can be kept sterile, which prevents disturbing contaminations.

Immediately after adding the spores, they are in close contact with the roots allowing detection of early host responses. Compared with the other two infection systems, transcriptional responses are very vigorous, which might be considered artificial in terms of strength as the plantlets are relatively small and rapidly overgrown by the pathogen. Nevertheless, as shown for the transcriptional induction of IG markers, expressional changes can be studied in both roots and leaves. As an advantage, many plants are used in pools (approx. 100 per plate), which reduces fluctuations due to variations in infection usually observed in plant pathogen interactions [35]. Genome-wide –omics approaches are feasible with root material such as Microarray, RNA-sequencing or even cell-layer specific assays [12, 36]. Expressional changes in roots can be easily corroborated with adequate reporter-lines (e.g., GFP, GUS or luciferase) and dynamics of fungal spread can be visualized with the GFP-tagged *V. longisporum* strain. Root phenotypes are well observable and quantifiable through measuring primary root growth or lateral root development upon microbial challenge. Using the model organisms *V. longisporum* and *Arabidopsis*, the petri dish system is not suitable to investigate time-points upon 6 dpi as plants are progressively harmed and dying. Susceptibility tests at relatively early time points (1 to 2 dpi) are practicable as long as differences regarding WT are substantial as demonstrated for the *cyp79b2/b3* double mutant. Subtle differences in susceptibility are difficult to detect with this system. In this case, it is recommended to choose one of the other two infection systems as they are more sensitive in analysing fungal spread, like demonstrated with the tolerant *ERF105 OE*. Quantification of general disease symptoms, such as reduced biomass, is hardly practicable in petri dishes and you rely on the measurement of fungal DNA as the best parameter.

Since the system is small, it is barely adequate for larger *B. napus* seedlings. Recently, Behrens et al. [37] established another infection system on plates, where a brush was dipped in *V. longisporum* conidia suspension and used to distribute spores along roots growing onto an agar medium. This method might be considered as an alternative.

### Advantages and disadvantages of the in vitro infection system in plastic cups

A yet unpublished in vitro infection system in plastic cups was established to investigate infection events temporally between the petri dish system (hours post inoculation to 6 dpi) and the soil-based system (> 21 dpi). This novel system is easy-to-handle, not very space consuming and it can be kept germ-free from external contaminations. The experimental set-up needs 4–5 weeks to obtain results.

Expressional changes can be analysed in roots and shoots leading to results comparable to the petri dish system, although with lower induction values for some marker genes. Nevertheless, since roots are embedded in agar medium and not as easy to access as in petri dishes, confirmation of expressional changes in roots with GFP/LUC-reporter lines is more difficult here (pulling out the roots leads to injury, which might interfere with reporter gene expression).

Progression of *V. longisporum* infection is slower compared to the petri dish system reflecting more natural conditions. The spores are not in immediate contact with the roots, they have to germinate in the infection channel and fungal hyphae have to grow through the medium towards the roots. Most suitable time points to perform pathogenicity tests with different *Arabidopsis* lines range between 10 and 16 dpi. Beyond this time, *Arabidopsis* might be extensively colonized and overgrown, leading to difficulties in detecting differences between plant lines. However, the best time point should be defined through preliminary experiments, as it was exemplified for the *cyp79b2/b3* mutant. A loss-of-function impairing IG biosynthesis increased susceptibility, while a gain-of-function enhanced tolerance. This is in line with the other experiments of this study and previous publications [12, 13], altogether confirming the reliability of the new system in plastic cups and its applicability to analyse fungal propagation in different plant genotypes. Therefore, identification of differentially expressed genes in roots and shoots as well as susceptibility tests are doable in parallel underlining the substantial advantage of this system.

Regarding the larger *B. napus* seedlings, this infection system is also suited, but later time points (14 to > 20 dpi) are advisable for susceptibility tests, as fungal spread is delayed and weaker in comparison to *Arabidopsis*. Other brassicaceous model plants could also be applied here (e.g., broccoli).

Although *V. longisporum* and the devastating *V. dahliae* are closely related, they differ in the degree of host specialization. While the *V. longisporum* host range appears to be restricted to members of the *Brassicaceae* family, *V. dahliae* is pathogenic on a broad range of hosts comprising more than 200 plant species including many crop plants [1, 38]. This makes *V. dahliae* an economically central model root pathogen to study. Here, the plastic cup system offers promising opportunities for future research. It appears to be suitable for analysing at least the *Arabidopsis*—*V. dahliae* and tomato—*V. dahliae* pathosystems.

Interestingly, *Verticillium* reaches the foliage of *Arabidopsis* after inoculating at the roots without damaging the root tissue. qPCR analysis demonstrated a rapid colonization of the foliage in the agar-based in vitro systems (petri dishes, plastic cups), which reflects propagation via the xylem. In petri dishes, this is a very rapid process within 1 to 2 dpi. As the endodermis provides a barrier to restrict *V. longisporum* propagation to the vasculature [26, 36], the fungus might penetrate either through not fully developed tissues (i.e., meristematic/elongation zones), at sites where emerging lateral roots burst through outer cell-layers [39] or it may weaken the endodermal barrier actively. This observation in agar-based infection systems is new, since it has been assumed that *Arabidopsis* roots need to be wounded prior inoculation to facilitate *V. longisporum* invasion, as it is the case in the soil-based infection system.

### Advantages and disadvantages of the soil-based infection system

Since roots are damaged due to up-rooting, this opens a gate for *V. longisporum* to enter the vasculature directly, which makes this system a bit artificial. On the other hand, it could mimic natural conditions that injure roots, such as nematode feed [40]. However, of the three systems discussed, it is the one closest to natural conditions as plants grow in soil. The infection progression is slower compared to the other two systems and symptom development can be tracked (reduction in biomass/green leaf area). Analysing fungal colonization in stems is well suited to specify increased tolerance or susceptibility in extensive screenings of ecotypes in genome-wide association studies (GWAS) or transgenic plant collections (e.g., *At*TORF-Ex, [13]). Since infected plants are smaller than mock treated ones, the measurement of height might be an additional parameter to quantify symptoms [13]. Nevertheless, symptom extrapolation can lead to huge error bars due to individual variations. Symptom-derived results should always be validated through quantification of fungal DNA in leaves, which is by far the most reliable method. With *Arabidopsis,* best results are achieved here 21–28 dpi.

Applying this system, it takes 6–7 weeks to get results, it needs more space than the others and the conditions are considered to be semi-sterile only so that the bilateral interaction is not completely undisturbed. Pathogen-triggered expressional changes can be investigated in leaves, but hardly in roots. Since roots grow in soil, it is difficult to clean them sufficient enough for qRT-PCR analysis without the risk of reprogramming gene expression due to washing. This is a limitation, at least for the markers discussed. In leaves, the response to *V. longisporum* was weak and from the experience with the other two systems roots appear to respond stronger.

The *Arabidopsis*—*V. dahliae* pathosystem can also be studied with this method, but as *V. dahliae* colonizes aerial plant parts more efficiently compared to *V. longisporum* [26], other time points post inoculation might be considered.

Plantlets from *B. napus* can be successfully inoculated with *V. longisporum* similar to *Arabidopsis* using this technique as previously described by Singh et al. [41]. They infected 7 d old *B. napus* seedlings by up-rooting and subsequent root dip inoculation and found that the highest concentration of fungal DNA was present in hypocotyl stems. Thus, quantification of fungal DNA in *B. napus* seems to be more reliable with hypocotyls than with leaves (> 28 dpi).

### Indole-glucosinolates and their role against *Verticillium*

Genes associated with the formation of the Trp-derived secondary metabolites camalexin and IG were transcriptionally induced in *Arabidopsis* challenged with *V. longisporum*. Previously, the importance of these genes for defence was described [e.g., 12, 13, 36]. Genetic disruption of the complete pathway using a *cyp79b2/b3* mutant facilitates proliferation of fungi, such as biotrophic powdery mildews, the necrotrophic *Sclerotinia sclerotiorum*, and the beneficial endophyte *Serendipita indica* [19, 42, 43]. In line, this double mutant displayed significantly enhanced growth of both *V. longisporum* and *V. dahliae*. It is tempting to speculate that there is a broader effect of Trp-derived secondary metabolites against *Verticillium* species. Overexpression of *ERF105* activating IG marker gene expression reduced *V. longisporum* and *V. dahliae* spread. Although this points to an effect of IG in restricting *Verticillium* ssp. propagation, further studies are required to unravel the mechanism of how IG contribute to counteract these vascular root pathogens.

## Conclusion

Expressional induction of the IG biosynthesis is one important marker to evaluate *V. longisporum* infection. It is more crucial in roots than in shoots and dominant in the beginning of infection. Assays with mutants and overexpressors in the IG biosynthesis demonstrated that these are important secondary metabolites involved in the restriction of *Verticillium*. The given examples highlight the ability of the three described infection systems to further decipher root-microbe interactions, each with its specific pros and cons. Although the focus of this work was set on *V. longisporum*, the described infection systems can similarly be used for other soil-borne microorganisms and model plants, as briefly exemplified for *V. dahliae* and tomato. Additionally, the novel in vitro infection system in plastic cups might be feasible for studies with the soil-borne beneficial fungus *Serendipita indica*. Very recently, we applied *S. indica* successfully in the petri dish system to examine differentially expressed genes in roots [36]. This broadens the spectrum of the explained infection systems to study not only pathogenic but also mutualistic interactions helping to understand the complex associations in the rhizosphere where plant roots are constantly challenged.

## Supplementary Information

**Additional file 1: Figure S1.** Preparation of *Verticillium longisporum* inoculum and infection system in petri dishes. **Figure S2.** Overview of the soil based infection system in pots with *Arabidopsis*. **Figure S3.** Overview of the sterile in vitro infection system in plastic cups. **Figure S4.** Analysis of primer specificity for the organisms under investigation. **Figure S5.** Methodological details. **Figure S6.** Spread of *V. dahliae* in *Arabidopis* and tomato infected in the in vitro infection system in plastic cups. **Table S1.** Primer Oligomers used in this study.

## Data Availability

The datasets supporting the conclusions of this article are included within the article and its Additional file 1.

## Footnotes

1 Always use freshly harvested conidiospores to prepare inoculum and not (as it was described earlier [12]) frozen storage stocks. Storage stocks display reduced germination and viability, affecting reproducibility in independent experiments.

2 Avoid sugar in the agar medium as it will lead to excessive fungal growth after inoculation.

3 Keep the solidified petri dishes upside down in the fridge (4–10 °C) overnight before cutting. A chilled medium helps to prevent sliding after cutting. Avoid getting liquid or air under the agar medium while cutting. Otherwise, sliding occurs.

4 Do not autoclave at 121 °C as this will affect important organic compounds in the nutrition soil. Steaming at 80–100 °C for 20 min in an autoclave is fine to reduce microbial contaminations in the substrate to a very low rate.

5 Since *Arabidopsis* is sensitive to flooding, water only when there is no more standing water in the tray.

6 It is important to wash only the roots in water, while keeping the rosettes out of the water. If rosettes are too waterlogged, it may cause additional side effects due to hypoxic stress.

7 To facilitate plants’ repotting, a hole should be made in the soil. After inserting the roots, make sure to refill the holes very softly with soil and avoid pressing. Otherwise, stress symptoms can result from repotting (e.g., blueish leaves).

8 As alternative, for example Phytatray™ II (Sigma-Aldrich) or Magenta™ vessel (Sigma-Aldrich) can be used. Although they are more expensive, they are delivered ready-to-use germ-free.

9 Prepare 10–15% more cup systems as needed, because occasionally some seedlings do not grow properly.

10 Slightly scratch the solidified agar medium before placing the seeds on it. This interrupts the solidified skin at the surface, allows the seeds to soak with water and facilitates the radicle to penetrate the agar medium.

11 As it is obligatory to ensure gas exchange, take Leukopor^®^ or something similar.
