# Supplementary material for: In-depth evaluation of root infection systems using the vascular fungus Verticillium longisporum as soil-borne model pathogen
Source: Plant Methods. 2021 Jun 5;17:57. doi: 10.1186/s13007-021-00758-x (PMC8178838; doi:10.1186/s13007-021-00758-x)
Supplement: Supplementary file 1 — Additional file 1: Figure S1. Preparation of Verticillium longisporum inoculum and infection system in petri dishes. Figure S2. Overview of the soil based infection system in pots with Arabidopsis. Figure S3. Overview of the sterile in vitro infection system in plastic cups. Figure S4. Analysis of primer specificity for the organisms under investigation. Figure S5. Methodological details. Figure S6. Spread of V. dahliae in Arabidopis and tomato infected in the in vitro infection system in plastic cups. Table S1. Primer Oligomers used in this study. [file 13007_2021_758_MOESM1_ESM.pdf]

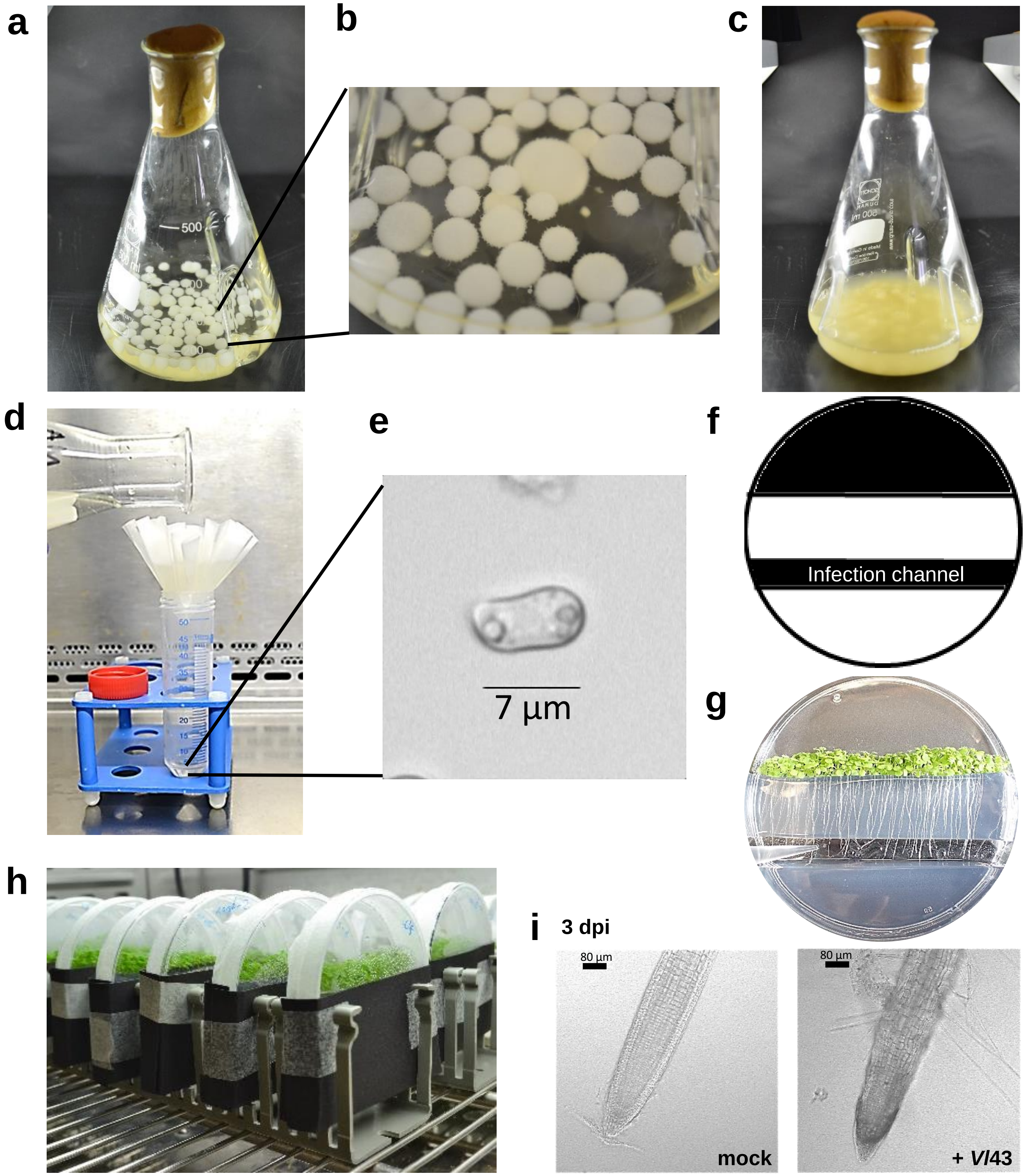

Fig. S1: Preparation of *Verticillium longisporum* inoculum and infection system in petri dishes. **a** / **b** The liquid PDB medium was inoculated with conidiospores and white mycelial balls appeared after 7 to 10 days under continuous shaking. **c** 4-5 days after the PDB medium had been replaced by liquid CDB medium, the supernatant became yellow-greyish due to the formation of new conidiospores. **d** Spores were purified through folded filter paper into a 50 ml tube. **e** Characteristic long-drawn conidiospore under the microscope. **f** Black areas in the scheme were removed from the solidified agar-medium with a scalpel. **g** 50-100 *Arabidopsis* plantlets were cultivated per plate and roots were inoculated via adding the conidiospore solution into the infection channel. **h** The Leukopor® sealed plates were placed vertically and root areas were covered with black papers to darken roots and soil-borne fungus. **i** Infected roots (+ *Vl*43) appeared different from mock treated ones under the microscope (3 dpi).

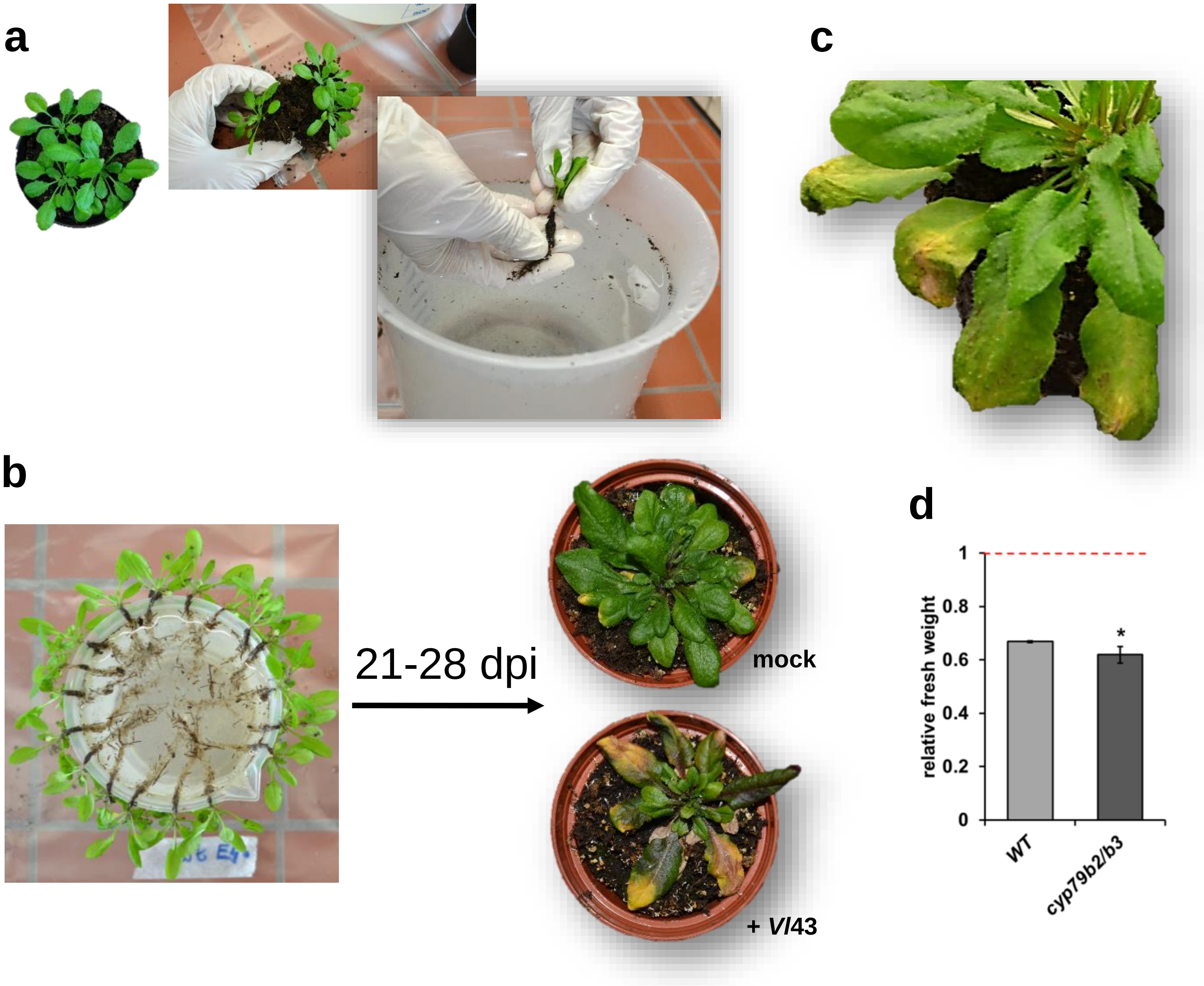

Fig. S2: Overview of the soil-based infection system in pots with *Arabidopsis*. a 3-4 *Arabidopsis* plants were cultivated per pot in a soil-sand mixture. To moderate phenotypical and individual variations, which might influence experimental outcome, plenty of plants were cultivated and just plants of similar size were selected for the experiments. 21 d old plants were prepared for "root dip inoculation": Carefully turning the pots without destroying the rosettes, excavating of single plants and washing of roots in a 5-liter jar. Only roots were gently washed with one hand, while the rosettes were kept out of the water with the other hand. This procedure injured roots, which facilitated *V. longisporum* to penetrate. **b** All plantlets were prepared and temporarily stored hanging on a small cup with the roots in water and the rosettes outside. After all plants were prepared like this, roots were rapidly transferred into petri dishes containing either a spore or mock solution. This ensured the same infection start time for all plants. Similar to the preparation shown in photo (b), just the roots were hanging in the spore solution, while the rosettes did not contact the solution. After a 60 min incubation, plants were repotted into single pots containing soil. 21-28 dpi, observable symptoms developed in infected plants (+ *Vl*43) compared to mock treated ones (demonstrated in the photos with *Arabidopsis* WT plants, 28 dpi). **c** Enlarged section of yellowish and necrotic leaves from *Vl*43-infected *Arabidopsis* WT (28 dpi). **d** Differences in infection progression between plant lines can be quantified either by fungal DNA measurement (like it is exemplified in the main figures) or via development of symptoms. Here, relative fresh weight of WT and *cyp79b2/b3* was determined (21 dpi). Fresh weight of mock controls from both lines was set to 1 (red dashed line) and infected samples are given relative to mock. Both infected WT and *cyp79b2/b3* plants showed a reduced fresh weight compared to their mock samples. Relative fresh weight of *cyp79b2/b3* was even more reduced than the one of WT (n = 15 each, ± SEM, student's t-test relative to WT, * $p \leq 0.05$).

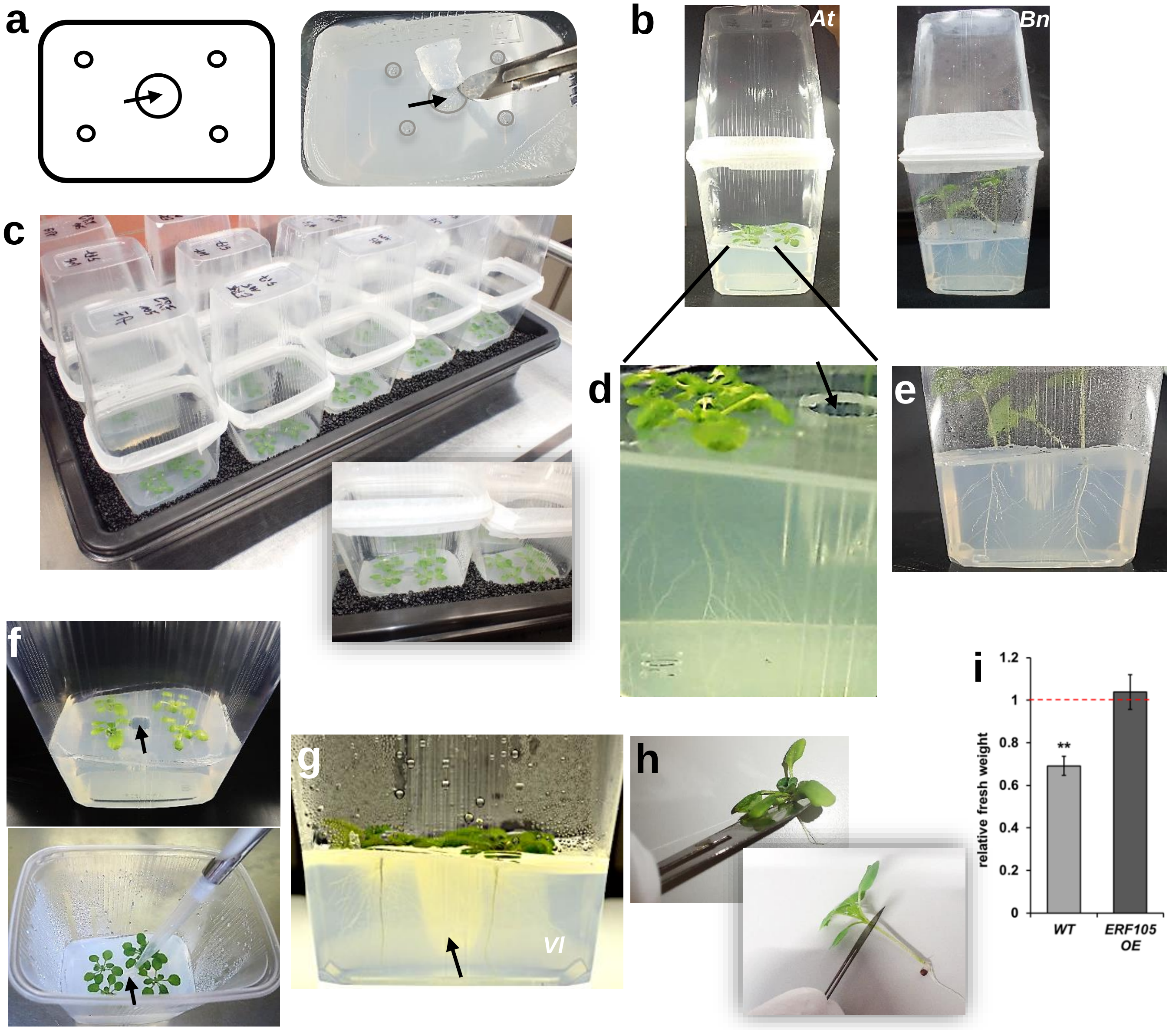

Fig. S3: Overview of the sterile *in vitro* infection system in plastic cups. In all pictures, the arrows indicate the infection channel. All steps were carried out in a laminar flow hood with sterile equipment. **a** Separating plastic layer as scheme (left; illustrates the prefabricated holes) and as photo (right). The separating layer lay on the agar-medium and the infection channel was cut in the centre hole (photo shows the removal of the agar block cut out with a scalpel). In each of the four smaller holes, one seed was placed after scratching the solidified medium. **b** The lower plastic cup with the plants growing in agar medium was covered with a second and inverted cup as lid. Closure with Leukopor® allowed gas exchange. Medium for *Arabidopsis* (*At*, left) filled approx. ⅓ of the lower cup, while more medium was needed for *Brassica napus* (*Bn*, right; approx. ½ - ⅔ of the lower cup). **c** In order to darken the roots, the lower parts of the cups were embedded in granulate (black split, 2-3 mm). **d** Enlarged section with *Arabidopsis*. The separating layer can be seen that prevents a direct contact of the leaves with the fungus after root inoculation. Therefore, all fungal DNA detectable in leaves derived solely from fungal spread within the plant from root to shoot. **e** Enlarged section with *Brassica napus*. **f** Inoculation of 21 d old *Arabidopsis* seedlings by adding a solution containing *V. longisporum* conidiospores into the infection channel. **g** Several days after inoculation, mycelium (*Vl*) became visible in the medium. **h** Whole rosettes were harvested with a scalpel from *Arabidopsis* (upper left). Roots were carefully pulled out of the medium and blotted with paper towel to remove agar remains. From *Brassica napus*, all leaves were harvested but not the stems (lower right). **i** Differences in infection progression between plant lines can be quantified either by fungal DNA measurement (like it is exemplified in the main figures) or via development of symptoms. Here, relative fresh weight of WT and *ERF105 OE* was determined (12 dpi). Fresh weight of mock controls from both lines was set to 1 (red dashed line) and infected samples are given relative to their mock. While infected *ERF105 OE* plants showed almost mock-like fresh weight, fresh weight of infected WT significantly decreased (n = 15 each, ± SEM, student's t-test relative to mock, ** $p \leq 0.01$).

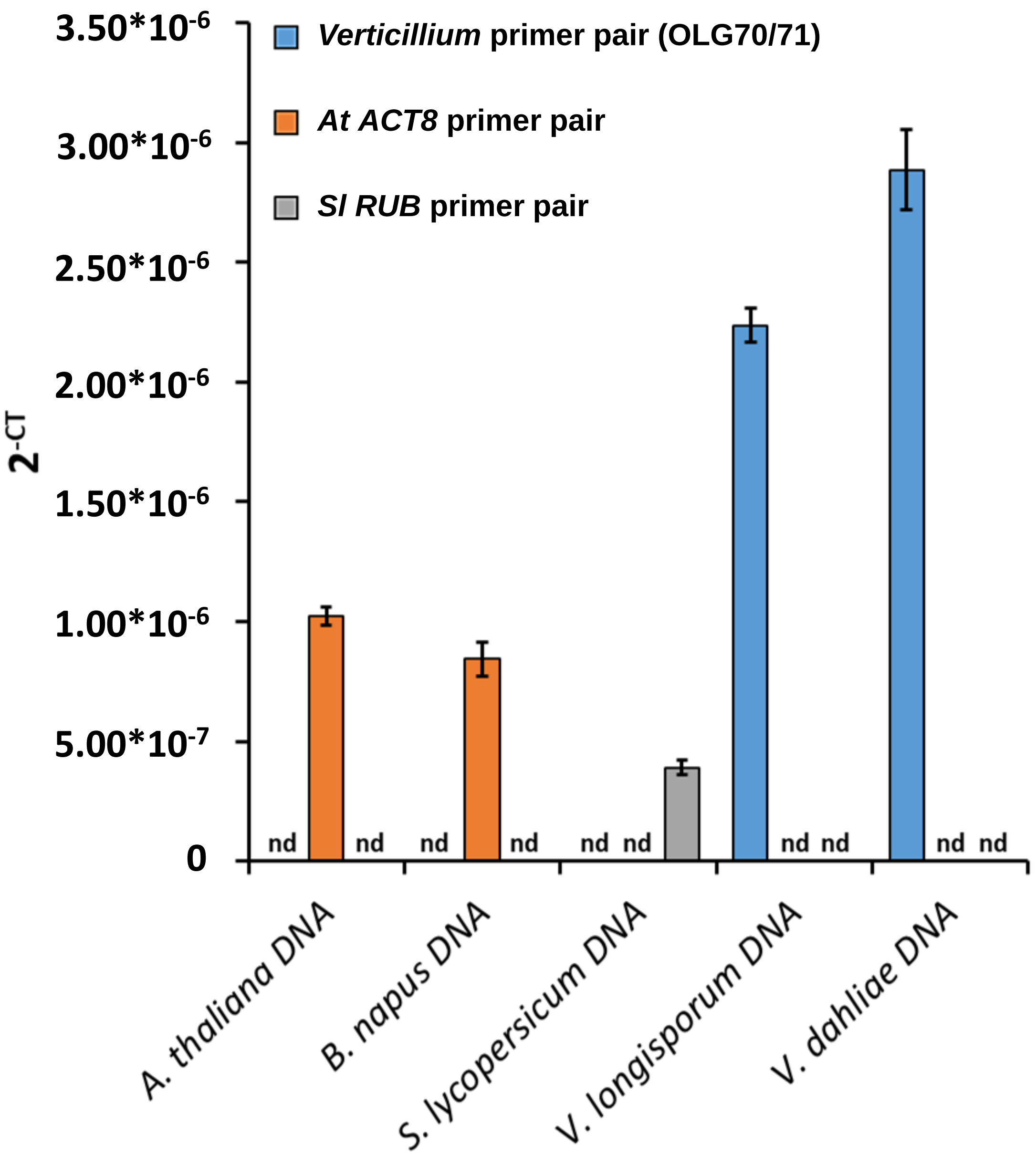

Fig. S4: Analysis of primer specificity for the organisms under investigation. Plants: genomic DNA was isolated from **uninfected** *A. thaliana* (*At*) or *B. napus* (*Bn*) leaves or *S. lycopersicum* (*Sl*) stem segments. Fungi: genomic DNA was isolated from freshly harvested conidiospores from either *V. longisporum* or *V. dahliae*. 100 ng of each genomic DNA extract served as template in qPCR with either *At ACT8*, *Sl RUB* or *Verticillium* specific primers (OLG70/71). 2^{-CT} values are given on y-axis (n = 3 each, ± SD). *At ACT8* primers led to amplification in DNA extracts of the two related species *A. thaliana* or *B. napus*, but not in *S. lycopersicum*, *V. longisporum* or *V. dahliae* DNA extracts. Therefore, *ACT8* primer pair can be used for normalization in infection experiments with *A. thaliana* and *B. napus*, respectively. *Sl RUB* primers led to an amplicon specifically with the *S. lycopersicum* DNA extract. The *Verticillium* primer pair amplifies a specific *Verticillium* fragment encoding parts of the 5S rRNA [8, 41]. The signal obtained with the *Verticillium* primer pair and *V. longisporum* / *V. dahliae* DNA extracts as template was strong, allowing the use of these primers to detect both *V. longisporum* and *V. dahliae* DNA. Using the *Verticillium* primer pair with *A. thaliana*, *B. napus* or *S. lycopersicum* genomic DNA as template resulted in almost no signal, although there was a bit of background noise interpretable as unspecific binding of the primers. This background noise with *Verticillium* primers in uninfected plant samples (mock) was sufficient enough to use it in the time course experiments to calculate the mock value and to normalize the infected samples to mock; nd = not detectable (defined as values < $3*10^{-10}$).

a

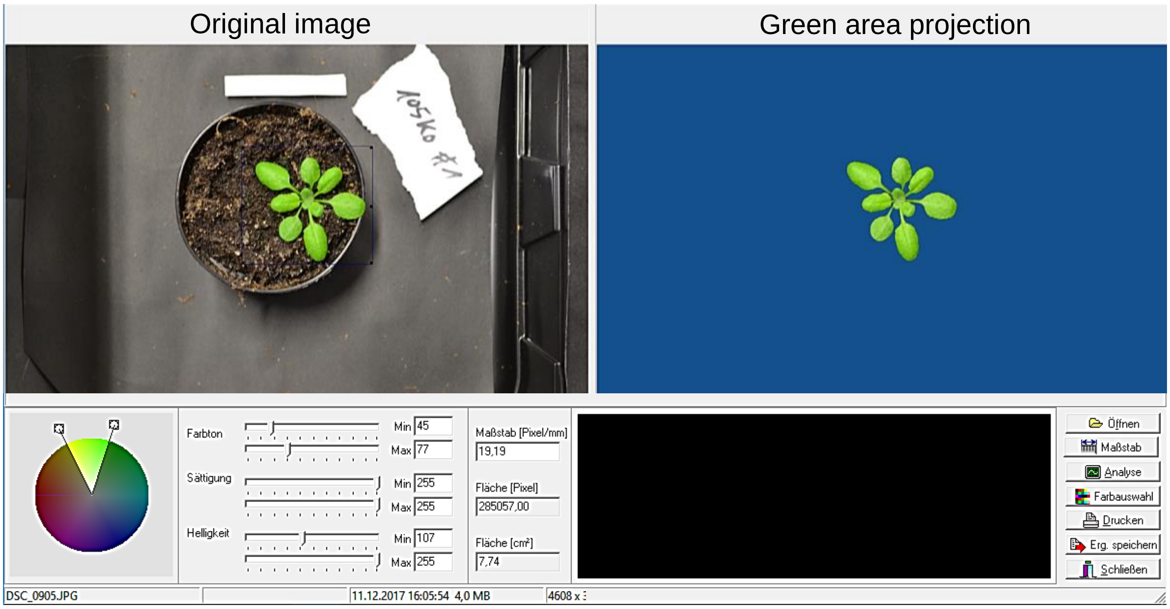

b

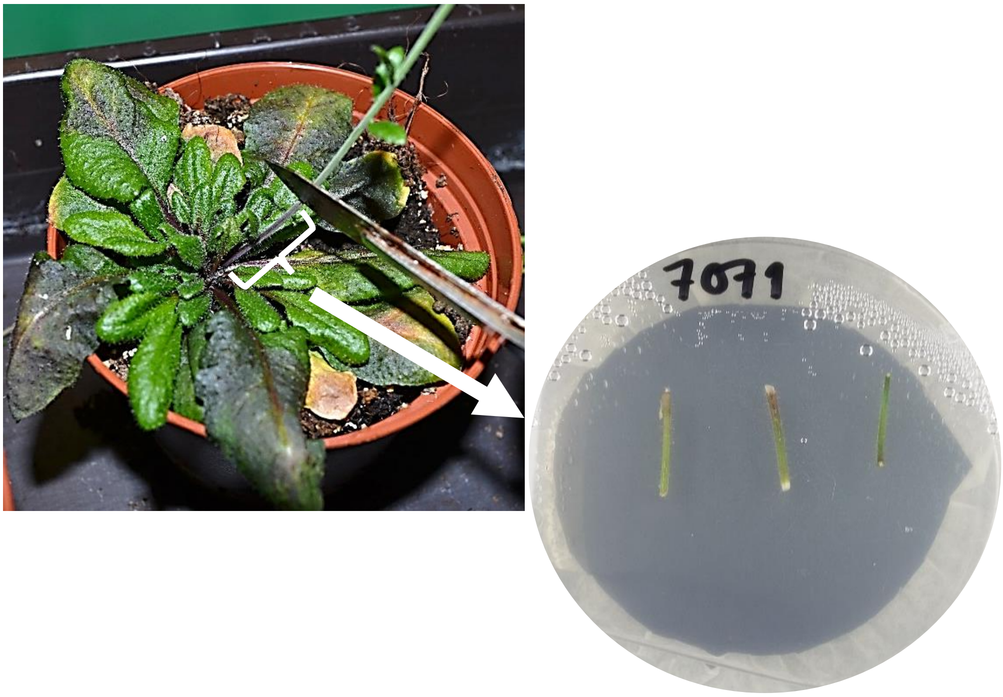

Fig. S5: Methodological details. a Infection symptoms were monitored by measuring the green leaf area on digital photos. The projected leaf area of the complete rosette was determined with the image analysis program BlattFlaeche® (Datinf GmbH, Tübingen, Germany). The settings were chosen so that only the green area was measured: the original picture on the left and the projected green leaf area on the right. If yellow leaf areas occurred, they were not projected. **b** From infected plants, 1 to 1.5 cm of inflorescence stem segments were cut from the bottom, surface sterilized and placed on solidified Potato Dextrose Broth (PDB) Agar. Fungal outgrowth initiated from the inside of the stems and 3-5 days later this became visible and could be evaluated or categorized (compare Fig. 2f).

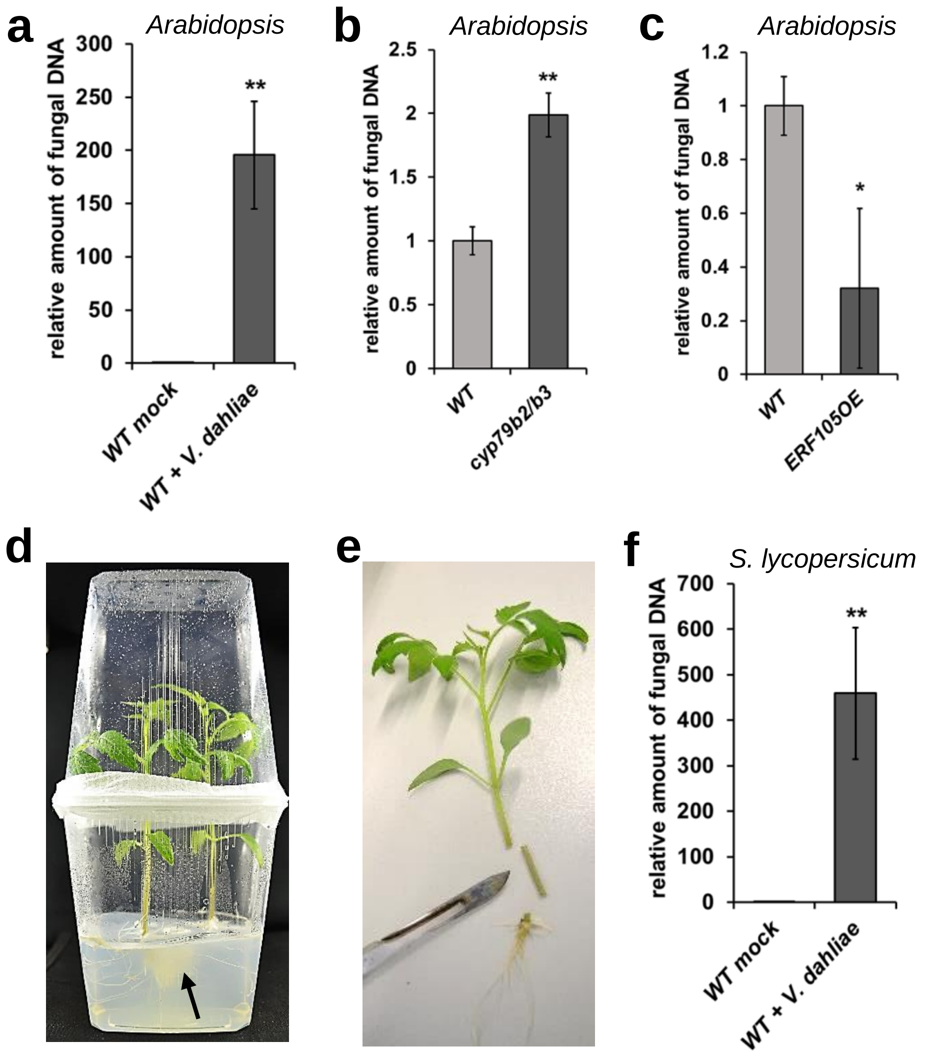

Fig. S6: Spread of *V. dahliae* in *Arabidopis* and tomato infected in the *in vitro* infection system in plastic cups. a 21 d old *Arabidopsis* WT plants were root inoculated with *V. dahliae* (JR2) and relative amount of fungal DNA was determined 12 dpi in leaves. Leaves from mock treated plants served as control. Values of infected samples are given relative to background noise in mock samples (set to 1) (*n* = 3 each, ± SD). **b / c** 21 d old plants from the *Arabidopsis* lines indicated were root inoculated with *V. dahliae* (JR2). Amount of fungal DNA was determined 12 dpi in leaves. Values are given relative to WT (set to 1) (*n* = 3 each, ± SD). **d** Tomato (*S. lycopersicum*) has grown well in the infection system in plastic cups. 12 d old plants were root inoculated in the infection channel (arrow) with *V. dahliae* (JR2). The photo shows 14 dpi. **e** 1 cm hypocotyl stem segments were cut with a scalpel from tomato. **f** Relative amount of fungal DNA was determined 14 dpi in tomato stem segments (see e). Values of infected samples are given relative to background noise in mock samples (set to 1) (*n* = 3 each, ± SD).

Fig. S6 statistics: student‘s *t*-test relative to mock (a, f) or WT (b, c), * $p \leq 0.05$, ** $p \leq 0.01$.

Table S1: Primer-Oligomers used in this study

Primer sequences for qRT-PCR and qPCR:

Gene ID	Primer Name	Sequence (5'->3')
AT4G39950	*CYP79B2* for	GAGACGACGCCGTATATATTTTATG
AT4G39950	*CYP79B2* rev	GGAGACACAATTCAAGTGTCCCAA
AT2G22330	*CYP79B3* for	ACCGGAAAGAGAGGATGTGCTG
AT2G22330	*CYP79B3* rev	CGCTAGCATCATGGTCGTTATCGC
AT5G57220	*CYP81F2* for	GAAGATGTTGACATGACAGAG
AT5G57220	*CYP81F2* rev	TGCTTAAACCGGTAAACTTC
AT5G51190	*ERF105* for	AGCTGCAAGAGGTTATGAC
AT5G51190	*ERF105* rev	TTCATCCTCCGTCACCTGTC
AT3G62250	*UBQ5* for	GACGCTTCATCTCGTCC
AT3G62250	*UBQ5* rev	GTAAACGTAGGTGAGTCCA
AT1G49240	*At ACT8* for	GGTTTTCCCCAGTGTTGTTG
AT1G49240	*At ACT8* rev	CTCCATGTCATCCCAGTTGC
S. lycopersicum RuBisCo	*Sl RUB for*	GAACAGTTTCTCACTGTTGAC
S. lycopersicum RuBisCo	*Sl RUB* rev	CGTGAGAACCATAAGTCACC
Verticillium 5S rRNA	*OLG70*	CAGCGAAACGCGATATGTAG
Verticillium 5S rRNA	*OLG71*	GGCTTGTAGGGGGTTTAGA
